# Best-worst scaling improves measurement of first impressions

**DOI:** 10.1186/s41235-019-0183-2

**Published:** 2019-09-23

**Authors:** Nichola Burton, Michael Burton, Dan Rigby, Clare A. M. Sutherland, Gillian Rhodes

**Affiliations:** 1 ARC Centre of Excellence in Cognition and its Disorders, School of Psychological Science, The University of Western Australia, 35 Stirling Hwy, Crawley, WA 6009 Australia; 2 School of Agriculture and Environment, The University of Western Australia, Crawley, WA Australia; 3 Economics, School of Social Sciences, University of Manchester, Manchester, UK

## Abstract

A common goal in psychological research is the measurement of subjective impressions, such as first impressions of faces. These impressions are commonly measured using Likert ratings. Although these ratings are simple to administer, they are associated with response issues that can limit reliability. Here we examine best-worst scaling (BWS), a forced-choice method, as a potential alternative to Likert ratings for measuring participants’ facial first impressions. We find that at the group level, BWS scores correlated almost perfectly with Likert scores, indicating that the two methods measure the same impressions. However, at the individual participant level BWS outperforms Likert ratings, both in terms of ability to predict preferences in a third task, and in terms of test-retest reliability. These benefits highlight the power of BWS, particularly for use in individual differences research.

**Electronic supplementary material:**

The online version of this article (10.1186/s41235-019-0183-2) contains supplementary material, which is available to authorized users.

## Significance

We quickly form impressions about the people we see based on their faces. Although these impressions are not necessarily accurate, they have broad implications - for instance, a person’s facial appearance predicts their rate of pay, their romantic success, and even their likelihood of a criminal conviction. It is therefore important to understand these powerful facial impressions. However, our ability to investigate these impressions is limited by the methods by which they are captured. Currently, researchers typically ask participants to rate their impressions of faces on a numeric scale. This method is well-established in psychological research but the resulting ratings can be biased, noisy and time-consuming. Here we demonstrate that an alternative method, best-worst scaling, allows us to more reliably capture participants’ facial impressions. This method will therefore make it easier to explain those impressions and discover their social impact. Our findings will improve face perception research, will help investigation of special populations, and can be used broadly across a range of applied vision topics.

## Best-worst scaling as an alternative to Likert ratings in first impressions research

Many important research questions in psychology require us to measure subjective impressions. For instance, we may be interested in how an anorexic person perceives bodies, or whether a child with autism is sensitive to the intensity of facial expressions. A relatively new area of research that almost exclusively uses this type of measurement is the study of facial first impressions. This research focuses on the trait impressions that we form within seconds of seeing a face. These facial first impressions do not necessarily reflect a person’s true nature: not all studies find evidence that first impressions accurately predict personality traits or behaviour, and where accuracy is found, effects are generally small (see Bonnefon, Hopfensitz, & De Neys, 2015; Todorov, Funk, & Olivola, 2015; Todorov, Olivola, Dotsch, Mende-Siedlecki, & Fiske, 2015 for review and discussion). Nevertheless, these impressions have important social and economic consequences: for example, they can predict individuals’ rates of pay and promotion, political success, and even criminal sentencing (see Olivola, Funk, & Todorov, 2014; Todorov, Olivola, Dotsch, & Mende-Siedlecki, 2015 for reviews).

A common approach to measuring subjective impressions is to use Likert ratings (see work cited in a review by Todorov, Olivola, Dotsch, & Mende-Siedlecki, 2015). Participants indicate the strength of their impression using a response scale with anchored endpoints. For instance, they might be asked to rate their impression of the attractiveness of faces on a scale from 1, “not at all attractive”, to 9, “extremely attractive.” This method is simple and straightforward for the experimenter, but is prone to a number of response biases and difficulties. First, participants are often reluctant to use the extremes of the scale (Baumgartner & Steenkamp, 2001; Weijters, Cabooter, & Schillewaert, 2010), which leads to responses being compressed with reduced differentiation between items. Second, participants may vary in how they use a scale. For instance, one participant may assign the highest point on the scale to the most attractive face in the set, while another may reserve that point for an imagined face that is much more attractive than the presented materials, compressing the range of values assigned in the task. Researchers often include instructions aimed at mitigating these biases (for instance, asking participants to use the full range of the scale in their ratings, and showing them the items in a set before they are rated). However, it is difficult to establish the effectiveness of these instructions, since response bias cannot be disentangled from participants “true” impressions of the items. Third, participants are not required to discriminate between items, and (at the extreme) are able to give all items the same rating. Finally, effective use of the scale requires that participants remain consistently calibrated throughout the task. Maintaining this calibration is cognitively demanding, as participants must remember their responses to previous items (see Clark, Howard, Woods, Penton-Voak, & Neumann, 2018; Kiritchenko & Mohammad, 2017a, 2017b for similar arguments). Together, these difficulties introduce biases and error that can reduce the validity and reliability with which impressions are measured.

Reliability of measurement is a particular concern for individual differences research, because the correlation possible between two measures is limited by the reliability of each measure (Spearman, 1904). In the case of facial first impressions research there is a growing interest in individual differences, because these can explain at least as much variation in facial impressions as shared taste (Germine et al., 2015; Hehman, Sutherland, Flake, & Slepian, 2017; Hönekopp, 2006; Kramer, Mileva, & Ritchie, 2018). To better understand what drives these stable idiosyncratic individual differences in first impressions, it is critical to use the most reliable measurement methods available.

**Fig. 1:**
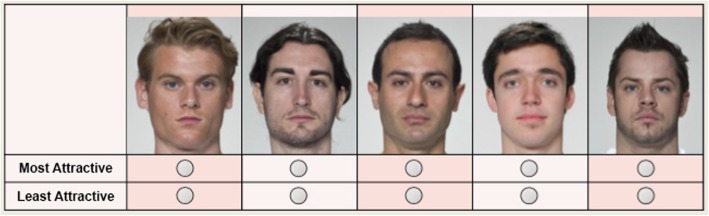
An example of a best-worst scaling (BWS) trial. Participants view a subset of the faces to be rated, and select the “best” (in this case, most attractive) and “worst” (in this case, least attractive) from the subset. This “best”/“worst” decision is easy to understand, naturalistic and relies only on the faces presented in the current trial, with no need to remember previous responses. These faces, from the Face Research Lab London Set (DeBruine & Jones, 2017), are for illustration purposes only, and were not used in the studies reported here

Best-worst scaling (BWS) is a promising alternative to Likert ratings. On each trial of BWS, participants are presented with a small subset of items (typically four or five) and select the “best” and “worst” (most and least attractive, etc.) items from that set (see Fig. 1 for an example of a trial). Items appear in multiple trials in varying combinations. A participant’s responses across all of the trials in the task reflect their preferences or impressions. For example, the face that the participant considers to be the most attractive in the entire stimulus set is expected to be selected as “most attractive” in every subset in which it appears, and likewise the face that the participant considers to be least attractive in the entire stimulus set is expected to be selected as “least attractive” in every subset in which it appears. The more attractive a face is to the participant, the more trials on which it will be selected as “most attractive” and the fewer trials on which it will be selected as “least attractive”. The rankings can be estimated either with simple scoring algorithms (see Hollis, 2018; Hollis & Westbury, 2018) or by estimating regression models (Louviere, Flynn, & Marley, 2015).

Best worst scaling is an extension of Thurstone’s method of paired comparisons (Thurstone, 1927), in which participants select the preferred option from every possible pair of items. Like the method of paired comparisons, BWS avoids many of the problems associated with the use of Likert ratings. Participants are required to differentiate between the items in the set, and because no response scale is used there are no issues of differences in scale use or interpretation between participants. Additionally, participants are not required to calibrate their responses to the range of variation in the set or to remember previous responses - each response depends only on the items presented in that trial. The advantage of BWS over the method of paired comparisons is that more information is provided in each trial, significantly reducing the number of trials required. In the method of paired comparisons, rating just 50 items requires 1225 trials, whereas in the BWS experimental designs presented here, the number of trials is equal to the number of items in the set (i.e. 50 trials to score 50 items), making BWS practical and cost-effective.

Importantly, Kiritchenko and Mohammad (2017a) have recently shown that BWS produces more reliable annotations of verbal data than Likert ratings, both when measured at the group level, and when considering the consistency of an individual’s annotations over time. These findings suggest that BWS may also be a preferable alternative to Likert ratings in tasks that involve quantifying participants’ subjective impressions of visual materials, such as faces. However, the use of BWS in experimental psychology has so far been limited. The existing applications in the psychology literature concern only verbal materials - either for establishing semantic norms (Hollis, 2018; Hollis & Westbury, 2018) or for ranking value statements in the personality literature (Lee, Soutar, & Louviere, 2007, 2008). To the best of our knowledge BWS has not yet been investigated as a potential method in the study of visual perception, and in particular has not been used to measure facial first impressions.

Here, we validate the use of BWS for quantifying facial first impressions by comparing BWS scores to Likert scale ratings of the same faces. First, we examine whether participants make the same judgement when they used the two methods, and find that BWS scores correlate strongly with Likert ratings at the group level. Given that the two methods appear to tap the same impressions, we next investigate potential benefits of the BWS method. If BWS is less subject to problems with response scale use, then we would expect BWS scores to better reflect participants’ impressions and therefore show better validity and reliability than Likert ratings. We find that BWS scores are a better predictor of individual participants’ preferences on a separate criterion task compared to Likert ratings, indicating improved validity for BWS ratings. Finally, we demonstrate that BWS scores show better test-retest reliability than Likert ratings. For the interested reader, we provide a common-sense guide to BWS plus R scripts that manage the processes of designing, running and scoring a BWS task in Additional file 1.

## Study 1

Participants rated the attractiveness of a set of 30 faces using both Likert ratings and BWS. We predicted that if participants’ responses when completing the BWS task reflect the same impressions as they do when making Likert ratings, then the scores assigned to the faces by the two methods (calculated across participants) should be strongly correlated. We also aimed to test the validity of the two methods by including a “criterion” task at the end of the testing session. In this task, participants ranked sets of three previously rated faces in order of attractiveness. If the scores produced by the BWS and/or Likert methods accurately reflect a participant’s impressions, then these scores should be able to predict the participant’s subsequent ranking of the faces in the criterion task. We predicted that BWS scores would be less subject to errors and biases in responding than Likert ratings, and that they should therefore better predict participants’ behavior in the criterion task.

### Methods

#### Participants

Participants were recruited from Amazon Mechanical Turk (MTurk). We recruited only participants who resided in the USA, and only participants using a desktop or laptop computer were able to take part. Following the initial demographic questionnaire, we screened out participants who were non-Caucasian (who could be subject to an other-race effect, see Meissner & Brigham, 2001, for a review) or older than 50 years (after which face identification ability has been shown to decline below young adult levels: Germine, Duchaine, & Nakayama, 2011). Participants screened from the study at this stage were paid US$0.05 for the time taken to complete the demographic questions. We also screened out any participants who failed any of three attention checks: a task in which participants had to identify the photograph containing an animal from two distractors in each of five sets, a multiple choice question about this task (“What were you looking for in the images above?”) and a question that participants were asked to leave blank.

Three-hundred and ninety-one participants completed the full task, which took approximately 10 min, and were paid US$0.70. We excluded the data on two participants who only pressed one key during the ratings task (indicating a lack of attention), giving a final sample of 389 participants (199 male, mean age = 32.4 years, SD = 7.4 years).

#### Materials

Thirty Caucasian male faces were selected from the Chicago face database (Ma, Correll, & Wittenbrink, 2015).

#### BWS design

For the BWS version of the task, we selected designs using Sawtooth Software’s *Lighthouse Studio* (Sawtooth Software, 2009). We specified a design with 30 items shown in 30 trials, such that each trial contained 5 items and each item was shown in 5 trials. Because there is no balanced incomplete block design (BIBD, the preferred design type for BWS tasks: see Guide in Additional file 2 for more information) with these parameters, we used Lighthouse Studio’s design algorithm to select designs that optimized balance (again, see Guide in Additional file 2 for more information). To avoid any unwanted dependencies between items, we selected 20 such designs and randomly allocated participants to a design.

#### Procedure

The task was presented online using Lighthouse Studio (Sawtooth Software, 2009). Participants first viewed the 30 faces one at a time, for 300 ms each, to familiarize them with the range of variation in the set. They were encouraged to consider how attractive/unattractive the faces were. Participants then completed the BWS and Likert blocks, with the order counterbalanced between participants. Finally, participants completed the criterion ranking task. Participants were asked to minimize distractions while completing the task and to wear glasses or other vision aids if required.

In the Likert block, participants were given the following instructions: “We are now going to ask you to rate the faces on a scale of 1 to 9, where 1 is Not at all Attractive and 9 is Extremely Attractive. We are interested in your impressions. There are no right or wrong answers. Please try to use the full scale.” Participants rated each face in turn; the face remaining on screen until the rating was selected. Each face was rated once, for a total of 30 trials.

In the BWS block, participants were given the following instructions: “We are going to show you sets of 5 faces, and ask you to select the face that is Most Attractive, and the one that is Least Attractive. There are several sets of these questions: this allows us to get a better understanding of which faces you find attractive, and which not.” Participants were shown five faces at a time, with the question: “Considering only these faces, which is the Most Attractive, and which is the Least Attractive?” The task began with two practice trials (using faces not present in the main face set) to familiarize participants with the procedure. Participants then completed 30 trials, with each face appearing in five of those trials.^1^

The last part of the testing session was the criterion ranking task. In each trial, participants were shown three of the faces previously seen in the Likert and BWS tasks. They were asked to order these faces from most attractive to least attractive. Participants completed six of these ranking trials (18 faces in total). The same 18 faces, arranged in the same sets, were given to all participants.

### Results and discussion

We began by examining the relationship between the group-level scores assigned to each face by the two methods. Likert scores were calculated as the mean rating given to each face. Reliability of these mean scores was calculated using Cronbach’s alpha, with individual raters treated as “items” in the analysis (Berry, 1991). Alpha in this analysis can be interpreted as the predicted agreement between this sample of raters and another sample of the same size. Alpha was equal to 0.93, indicating good reliability of the group-level Likert scores. BWS scores were calculated using the counts method (number of times a face was selected as most attractive minus number of times it was selected as least attractive). The scores given to each face by the two methods were very strongly correlated, Pearson’s *r*(28) = .99, *p* < .001. This finding indicates that participants were basing their responses on the same impressions in each condition.^2^

We then investigated whether BWS or Likert scores better predicted responses on the criterion task at the individual participant level. Participant-level BWS scores were calculated using the counts method. We then used rank-ordered logistic regression models to predict participants’ rankings of the faces in a criterion trial from either the BWS counts or Likert ratings that participants gave to those faces. We can compare the model fits by comparing Akaike’s bias-corrected information criterion (AIC_C_) values for each model. Models with an AIC_C_ difference of < 2 are considered equivalently good fits, while an AIC_C_ difference of > 10 indicates a substantial improvement in fit (Symonds & Moussalli, 2011). For each of the six criterion trials, the BWS scores better predicted participants’ rankings of the faces than the Likert scores (change in AIC_C_ > 10 in all cases: see Table 1). The BWS scores therefore show better validity than the Likert ratings as judged against this ranking criterion.

**Table 1:** Log-likelihoods and AIC_C_s for the rank-ordered logistic regression models predicting participants’ rankings for each criterion set from their BWS or Likert scores. Higher log-likelihoods, and lower values of Akaike’s bias-corrected information criterion (AICC) (i.e. closer to zero for both measures) indicate better model fit. Models using best-worst scaling (BWS) scores achieved better fit for each of the six criterion sets. This improvement is reflected by the difference between AIC_C_ values for the two models (*∆*_i_), which are > 10 for all criterion sets, indicating a substantial improvement in model fit (Symonds & Moussalli, 2011)

Criterion set	BWS		Likert		*∆*_*i*_
	Log-likelihood	AIC_C_	Log-likelihood	AIC_C_	
1	− 528.74	1059.49	− 562.19	1126.39	66.90
2	− 317.47	636.95	− 366.44	734.89	97.94
3	− 509.45	1020.91	− 563.55	1129.11	108.20
4	− 400.60	803.21	− 478.17	958.35	155.14
5	− 494.29	990.59	− 548.38	1098.77	108.18
6	− 489.88	981.77	− 561.11	1124.23	142.46

## Study 2

In study 2 we directly compared the test-retest reliability of BWS and Likert ratings. This approach (also utilized by Kiritchenko & Mohammad, 2017a) avoids the use of a criterion task (ranking) that is more similar to the BWS task (ranking) than that of the Likert ratings, which could potentially explain the better predictive power of BWS in study 1. Participants judged the distinctiveness of a set of faces in two sessions, separated by at least three days, with either Likert ratings or BWS (between participants). Facial distinctiveness is often reverse-scored as an approximation of the averageness of a face (Rhodes, Simmons, & Peters, 2005). This distinctiveness/averageness trait is an important aspect of face perception, since averageness contributes to attractiveness (Rhodes, 2006; Thornhill & Gangestad, 1999) and signals health (Lie, Rhodes, & Simmons, 2008). The reliability of distinctiveness ratings has previously been observed to be lower than other trait ratings (Foo, Simmons, & Rhodes, 2017), allowing greater scope to reveal any potential advantage of BWS. If participant-level BWS scores are more reliable than Likert ratings, then on average participants in the BWS condition should provide more consistent scores from time 1 to time 2 than participants in the Likert condition.

### Method

#### Participants

We recruited participants from MTurk who resided in the USA. Only participants using a desktop or laptop computer were able to take part. For this second study we recruited participants using a short qualifier task (paying US$0.10). This qualifier asked about participants’ age, gender, ethnicity and sexual orientation (orientation information not used for this task). We also included the three attention checks from study 1. Participants who passed the attention checks could then be invited to participate in further studies for which they met the demographic criteria. We chose this recruitment method because it is more transparent for MTurk participants than screening them after they have started a task, and it prevents participants from lying to meet the desired demographic criteria for a study. In the case of a test-retest experiment, it also has the benefit that participants received a similar invitation email to complete each part of the experiment, making the two sessions as equivalent as possible.

Three hundred and seventy-three participants completed session 1 of the study. Of these, 333 returned to complete session 2. Participants were compensated US$0.50 for participation in session 1 and US$1.00 for participation in part 2, with each session taking approximately 7 min. One participant was excluded because they pressed only one key in the ratings task (indicating a lack of attention), leaving a final sample of 166 participants in the Likert condition (57 male, mean age = 34.4 years, SD = 7.6 years) and 166 participants in the BWS condition (75 male, mean age = 34.2 years, SD = 8.3 years).

#### Materials

The same 30 faces used in study 1 were used in this study.

#### BWS design

For the BWS version of the task, designs were selected using Sawtooth Software’s Lighthouse Studio (Sawtooth Software, 2009) as described in study 1. Again, we selected designs with 30 items shown in 30 trials, such that each trial contained 5 items and each item was shown in 5 trials.

#### Procedure

Participants were randomly allocated to either the Likert or BWS condition. They were invited to return for a second testing session, in the same condition, 3 days after they completed their first testing session. The mean number of days between sessions was 5.7 days in the BWS condition (SD = 2.7 days) and 5.4 days in the Likert condition (SD = 2.1 days). Number of days between sessions did not differ significantly between conditions, *t*(312.98) = 1.28, *p* = .203. Both versions of the task were presented using Lighthouse Studio (Sawtooth Software, 2009). Participants were asked to minimize distractions while completing the task and to wear glasses or other vision aids if required.

Both tasks began with the following definition of distinctiveness: “Distinctiveness refers to how unusual a face is, compared to other faces. How much would this face stand out in a crowd? A face that stands out from other faces is distinctive. A face that blends into the crowd is not distinctive.”

The Likert condition followed the same procedure as in study 1, but this time participants were asked to rate the distinctiveness of each face on a scale from 1 (not at all distinctive) to 9 (very distinctive). We began by showing participants the 30 faces one at a time, for 300 ms each, to familiarize them with the range of variation in the set. Face order was randomized for each participant and in each session.

The BWS condition followed the same procedure as in study 1, but this time participants were asked to choose the most and least distinctive faces in each set. The task began with two practice trials (using faces not present in the main face set) to familiarize participants with the procedure. Faces were presented in different subsets for each participant and in each session.

### Results and discussion

We began by examining the relationship between the group-level scores given to the faces on this new trait, distinctiveness, by the participants in each condition. For this analysis we used only the responses from session 1. Likert scores were calculated as the mean rating given to each face. BWS scores were calculated using the counts method (number of times a face was selected as most attractive minus number of times it was selected as least attractive). The scores given to each face by the two methods were strongly correlated, Pearson’s *r*(28) = .86, *p* < .001. This finding again indicates that participants were basing their responses on the same impressions in each condition.

We then examined the consistency of participants’ responses across the two sessions. Individual participants’ BWS scores for each face were again calculated using the counts method. For each participant, we calculated the correlation between their session 1 and session 2 scores. Participants in the BWS condition had a mean Pearson’s *r* of .66 (SD = .18), whereas participants in the Likert condition had a mean Pearson’s *r* of .53 (SD = .22). Correlation coefficients are bounded, so we applied a Fisher transformation to statistically test the difference between the two conditions. The two-sample *t* test confirmed that scores in the BWS condition were significantly more strongly correlated between session 1 and session 2 than scores in the Likert condition, *t*(330) = 6.24, *p* < .001, Cohen’s *d* = 0.68. Thus, the BWS scores were more reliable than the Likert scores.

The analyses presented above demonstrate that BWS scores are more reliable than Likert ratings for individual participants. However, face ratings are also often used at the group level (for instance, taking a mean rating of a face’s distinctiveness ratings across participants that can then be related to other qualities of that face). In these cases, what is important is the reliability of the mean, group-level score. We therefore conducted a further analysis in which we compared the reliability of the group-level face scores from the two methods. Reliability was calculated as in study 1, using Cronbach’s alpha with individual raters treated as “items” in the analysis. The size of Cronbach’s alpha is positively related to the number of “items” - therefore our current sample size of 166 participants per condition would be likely to yield very high alpha coefficients for both conditions. For this reason, we calculated alpha for a range of smaller numbers of raters that might more typically be used to obtain face scores in a first impressions experiment: *N* = 8, 12, 20, 30, 40 and 50. For each sample size, we randomly sampled *N* participants from the full participant group and calculated alpha from their session-1 responses, repeating this process 50 times. The mean alpha values obtained for each *N*, in each condition, are reported in Table 2. Alpha values were consistently higher for BWS than Likert rating scores, particularly for typical sample sizes used in face perception studies, indicating that BWS scores are more reliable than Likert ratings at the group level.

**Table 2:** Mean Cronbach’s alpha calculated from 50 random samples of size *N* from each of the BWS and Likert conditions. Higher values of alpha indicate increased reliability for the face-level scores in that condition. *BWS* best-worst scaling

*N*	BWS	Likert
8	0.703	0.655
12	0.814	0.745
20	0.870	0.839
30	0.911	0.883
40	0.931	0.914
50	0.947	0.929
166	0.984	0.978

## Study 3

In Study 3, we aimed to replicate the findings of study 2 using a different face sample and a different trait judgment. We selected faces from the US 10 k database (Bainbridge, Isola, & Oliva, 2013), a database of images sourced from the Internet, which vary on many dimensions, including lighting, camera angle and facial expression. Increasingly, researchers study impressions of these “ambient” face images (Jenkins, White, Van Montfort, & Burton, 2011; Sutherland, Young, & Rhodes, 2017; Todorov & Porter, 2014), which more closely resemble the face images that people are likely to encounter online and in other media than highly controlled face sets. Our participants rated these faces on trustworthiness, a fundamental dimension of social perception (Sutherland et al., 2013; Todorov, Said, Engell, & Oosterhof, 2008).

Studies 1 and 2 were conducted using Lighthouse Studio (Sawtooth Software, 2009), which provides a conveniently streamlined process for study design, presentation and scoring. However, not all researchers may have access to Sawtooth. For this reason, we conducted study 3 using R (R Core Team, 2016) to build the study design and analyse the data, and presented our task online using Qualtrics survey software (Qualtrics, 2018). We include the R scripts used to run these processes in Additional file 1 as a reference for researchers who would like to build their own BWS task using these tools.

### Method

#### Participants

Participants were recruited from MTurk using the same pre-screening procedure as study 2. Two-hundred and sixty-three participants completed session 1 of the study. Of these, 202 returned to complete session 2. Participants were compensated US$0.90 for participation in session 1 and US$1.10 for participation in part 2, with each session taking approximately 7 min. Two participants were excluded because of missing data, leaving a final sample of 95 participants in the BWS condition (46 male, mean age = 43.0 years, SD = 12.5 years) and 107 participants in the Likert condition (54 male, mean age = 41.2 years, SD = 10.9 years).

#### Materials

Thirty-one faces were selected from the US 10 k database. This database contains images of adult faces obtained via google image search, cropped with an oval mask around the head area. We screened faces to ensure that they were Caucasian, forward-facing with direct gaze, and did not include any celebrities or other public figures.

#### BWS design

For the BWS version of the task, we selected a balanced incomplete block design (BIBD) using the *find.BIB* function in the R package *crossdes*. We specified a design with 31 items shown in 31 trials, such that each trial contained 6 items and each item was shown in 6 trials. In a BIBD with these parameters, every possible pair of items appears in exactly one trial. To reduce the effect of any higher-order dependencies in the trials, we created 8 BWS conditions, each one made by varying the assignment of face to item number (i.e. assigning face A to be item 1 in one version of the design, item 4 in the next version of the design, and so on). The details of these 8 BWS conditions, and the script used to create them, can be found in Additional files 1, 2 and 3.

#### Procedure

Participants were randomly allocated to either the Likert or BWS condition. They were invited to return for a second testing session, in the same condition, 3 days after they completed their first testing session. The mean number of days between sessions was 3.86 days in the BWS condition (SD = 0.64 days) and 3.83 days in the Likert condition (SD = 0.52 days). Number of days between sessions did not differ significantly between conditions, *t*(182.70) = 0.30, *p* = .763. Both versions of the task were presented online using Qualtrics survey software (Qualtrics, 2018). Participants were asked to minimize distractions while completing the task and to wear glasses or other vision aids if required.

The Likert condition followed the same procedure as in study 1, but this time participants were asked to rate the trustworthiness of each face on a scale from 1 (not at all trustworthy) to 9 (very trustworthy).

The BWS condition followed the same procedure as in study 1, but this time participants were asked to choose the most and least trustworthy faces in each set. Participants were randomly allocated to one of the eight BWS conditions.

### Results and discussion

We again began by examining the relationship between the group-level scores given to the faces by the participants in each condition. For this analysis we used only the responses from session 1. Likert scores were calculated as the mean rating given to each face. BWS scores were calculated using the counts method (number of times a face was selected as most attractive minus number of times it was selected as least attractive). The scores given to each face by the two methods were strongly correlated, Pearson’s *r*(29) = .98, *p* < .001. This finding once again indicates that participants were basing their responses on the same impressions in each condition.

We then examined the consistency of participants’ responses across the two sessions. As in study 2, we calculated individual participants’ BWS scores using the counts method. For each participant, we calculated the correlation between their session 1 and session 2 scores. Participants in the BWS condition had a mean Pearson’s *r* of .76 (SD = .18), whereas participants in the Likert condition had a mean Pearson’s *r* of .63 (SD = .23). Correlation coefficients were Fisher-transformed for parametric analysis. One outlier (Fisher-transformed *r* < 3 SD below the mean) was identified in the BWS condition, and one outlier was identified in the Likert condition: these outliers were removed from analysis.^3^ The Welch two-sample *t* test on the Fisher-transformed correlation coefficients confirmed that scores in the BWS condition were significantly more strongly correlated between session 1 and session 2 than scores in the Likert condition, *t*(195.74) = 5.17, *p* < .001, Cohen’s *d* = .73.

We also conducted the same group-level analysis as in study 2 to compare the reliability of face scores generated across the BWS or Likert groups at session 1. Again, reliability was calculated using Cronbach’s alpha with individual raters treated as “items” in the analysis. Mean alphas from 50 random samples of sample size *N* are reported in Table 3. Alpha values were consistently higher for BWS than Likert rating scores, particularly for typical sample sizes used in face perception studies, indicating that BWS scores are more reliable than Likert ratings at the group level.

**Table 3:** Mean Cronbach’s alpha calculated from 50 random samples of size *N* from each of the BWS and Likert conditions. Higher values of alpha indicate increased reliability for the face-level scores in that condition. *BWS* best-worst scaling

*N*	BWS	Likert
8	0.822	0.778
12	0.893	0.827
20	0.924	0.901
30	0.951	0.931
40	0.962	0.948
50	0.970	0.958
122	0.988	0.983

## General discussion

Here we present the first demonstration that BWS is not only an effective method of quantifying participants’ subjective impressions of faces, but may also be superior to Likert ratings. Our results demonstrate that the two methods tap the same impressions, measured at the group level, but that BWS scores better predict participants’ subsequent rankings of faces, and show better test-retest reliability, than Likert ratings. These reliability benefits mirror those found for verbal materials (Kiritchenko & Mohammad, 2017a), and make BWS an excellent option for individual differences research, which critically depends upon the use of reliable measures (Spearman, 1904). These findings are particularly promising given the increasing interest in individual differences in facial first impressions (Germine et al., 2015; Hehman et al., 2017; Hönekopp, 2006; Kramer et al., 2018).

As well as superior reliability for individual-level scores, we also showed that BWS produced more reliable group-level scores, particularly when the number of raters was small. BWS could therefore be useful in the efficient collection of group-level scores for a set of faces. It should be noted that the BWS design employed here, with as many trials as there are items, is optimised for participant-level scores (Orme, 2005). If only group-level scores are required then substantially fewer trials are needed. Future research might fruitfully investigate the shortest testing time and minimum number of participants required to obtain stable group-level BWS scores for a set of faces.

BWS may be particularly beneficial for more diverse samples of participants, beyond the typical adults tested here. There is increasing interest in facial first impressions in clinical populations (Ewing, Caulfield, Read, & Rhodes, 2015a; Sprengelmeyer et al., 2016; Trémeau et al., 2016) and in children (Cogsdill, Todorov, Spelke, & Banaji, 2014; Ewing, Caulfield, Read, & Rhodes, 2015b). For these populations it can be difficult to achieve large sample sizes and there may be additional noise in responses, making any gains in reliability particularly important. Special populations who have problems with memory and/or executive function may also have difficulty maintaining good calibration of the Likert scale. These groups may find the trial-by-trial nature of BWS easier to manage, potentially opening up new populations for research. Young children who do not yet have a strong understanding of a number line or subtle differences in degree may also be better able to manage the simpler “most/least” decisions required in BWS.

A potential limitation of BWS is that the scores are relative (indicating impressions of an item relative to other items of the set) rather than absolute. We can use BWS to compare two participants in terms of the relative scores that they give to two faces (i.e. participant 1 gave Jim a higher score than Bob, while participant 2 gave Bob a higher score than Jim). However, where Likert ratings might indicate a mean difference between participants (i.e. participant 1 gave a higher mean rating to the items in the set than participant 2), there are no mean differences between participants in BWS. In some cases, failure to capture such differences would be beneficial, as when mean differences are caused by non-meaningful differences in scale use between participants (for instance, a willingness, or lack thereof, to assign socially undesirable scores to faces). However, in other cases, there may also be a meaningful component to these differences: for instance, one person may genuinely be more trusting than another or have a higher standard for attractiveness (Hönekopp, 2006).

If we are interested in mean differences, then the BWS approach can be augmented with “anchor” questions that aim to determine some fixed point on each participant’s set of scores that can be compared between participants (e.g. the level of trustworthiness at which a participant would lend a person money: Lattery, 2011). However, many research questions do not require this anchoring information: for instance, testing children’s ability to identify traits observed by adults (e.g. Ewing et al., 2015b), measuring the level of agreement between individuals’ rankings of faces (e.g. Hönekopp, 2006), or investigating the extent to which impressions reflect real-world characteristics of the pictured persons (e.g. Rule, Krendl, Ivcevic, & Ambady, 2013). For these questions, the non-anchored version of BWS presented here is ideal.

In the studies presented here we compared BWS and Likert ratings for impressions of three traits: trustworthiness, distinctiveness, and attractiveness. We see no compelling reason why other traits that are typically measured with Likert ratings should not also be appropriately measured using BWS. Nevertheless, it may be useful to conduct similar comparisons for other trait judgements: for instance, it may be the case that BWS gives greater advantages for measurement for some traits (e.g. dominance, a complex and context-dependent trait: Sutherland, Oldmeadow, & Young, 2016) than for others (e.g. perceived age). Because each BWS trial includes multiple faces, it may be easier for participants to maintain a single, consistent interpretation of the trait to be rated in the BWS paradigm, rather than varying their interpretation stimulus-by-stimulus (Hollis & Westbury, 2018). Following this line of reasoning, complex trait judgements that involve synthesising several cues might benefit more from the BWS method.

We have demonstrated that BWS is an advantageous method for quantifying participants’ impressions of faces. BWS may also be a fruitful method for other research areas that use similar materials: for instance, object perception or body perception. More generally, given our findings and those from research in the areas of language processing (Hollis, 2018; Hollis & Westbury, 2018) and personality (Lee et al., 2007, 2008), we recommend that researchers in any area of experimental psychology should consider whether BWS might be an appropriate substitute for Likert ratings, especially in individual differences research where reliability is particularly critical.

## Conclusions

In summary, we have demonstrated that BWS is a highly promising method for quantifying participants’ subjective impressions of visual materials - for instance, for obtaining scores of participants’ facial first impressions. BWS compares favourably to Likert ratings, the current standard method of measuring facial impressions: it better predicts participants’ rankings of faces and shows greater test-retest reliability. It is also less cognitively demanding than Likert ratings, and may allow us to test a more diverse range of participant groups. BWS therefore promises to be a useful tool for understanding the determinants of facial first impressions, which have a broad and substantial social impact.

## Additional files

Additional file 1: R scripts used to generate the design and sort and score the data of Study 3, with annotation: intended as a template to build future BWS studies. (ZIP 15 kb)

Additional file 2: A beginners' guide to the process of designing, running and scoring a Best-Worst Scaling task. (DOCX 107 kb)

Additional file 3: csv files containing data and design details for Study 3: required by the R scripts in Additional File 2. (ZIP 58 kb)

## Data Availability

The datasets analysed during the current study are available from the corresponding author on reasonable request.

## Footnotes

1 At the end of the BWS block participants also completed a two-alternative forced choice attractiveness judgement task using the same 30 faces, which was included for a separate study (data not analysed here).

2 Group-level scores in a BWS analysis are often produced using a conditional logistic regression model that estimates the probability of an item being selected as “best” or “worst” (Louviere et al., 2015). When BWS scores were calculated using this method the relationship between BWS and Likert scores did not change, Pearson’s *r*(28) = .99, *p* < .001.

3 Note that the conclusions of the analysis do not change if these outliers are retained: *t*(197.68) = 4.95, *p* < .001, Cohen’s *d* = .70.
