# Supplementary material for: Best-worst scaling improves measurement of first impressions
Source: Cogn Res Princ Implic. 2019 Sep 23;4:36. doi: 10.1186/s41235-019-0183-2 (PMC6757072; doi:10.1186/s41235-019-0183-2)
Supplement: Supplementary file 2 — A beginners' guide to the process of designing, running and scoring a Best-Worst Scaling task. (DOCX 107 kb) [file 41235_2019_183_MOESM2_ESM.docx]

**Running a Best-Worst Scaling (BWS) Task**

In each trial of a BWS task, the participant views a small subset of the faces to be scored. The participant selects which face in the set is the highest on some trait or quality, or “best”, and which is the lowest on that trait or quality, or “worst”.

Here we outline how to design, run and score a BWS task. We use examples from our three studies of facial first impressions, and refer to “faces” throughout, but any item type (other images, words, statements, etc.) can be used.

For more information on how to run this process using open-source packages in R, we recommend the excellent reference by Aizaki, Nakatani, and Sato (2014).

**Design**

It is up to the researcher to determine how many faces appear in each trial. Orme (2005) suggests that there is little gain in precision from showing more than 5 items per trial.

The researcher then needs to select which faces will appear in each trial. The recommended approach is to find a Balanced Incomplete Block Design (BIBD). A BIBD is arranged so that every face appears in the same number of trials, and every pair of faces appear together in the same number of trials. It is referred to as “incomplete” because any given trial contains fewer than the total number of faces. BIBDs for a given combination of parameters (i.e. number of faces, number of trials, and number of faces per trial) can be looked up in reference lists (for instance, Di Paola, Seberry, & Wallis, 1973) or generated in R (e.g. from the crossdes R package; see script S01 for a working example).

The benefit of a BIBD is that it reduces the number of unintended dependencies in the design that might bias the resulting ratings. For example, if the two most attractive faces are only ever seen together (i.e. in the same trials), then the most attractive will always be selected as “best”, biasing the attractiveness rating of the second-most attractive face downwards.

Note that it is not mathematically possible to generate a BIBD for every combination of parameters (number of items, trials, and items per trial; just as it is not possible to fully counterbalance an experiment given certain numbers of conditions and participants). If a BIBD for the desired combination of parameters does not exist, then one may be obtained by slightly altering the parameters (for instance, by changing the exact number of face items).

Alternatively, the researcher can attempt to find a design that approximates a BIBD as closely as possible with the given parameters.

Finally, whether or not a BIBD is used, we recommend that researchers select several different versions of their chosen design and randomly allocate participants to one of these versions. This procedure will further reduce the possibility of accidental dependencies between items.

For Studies 1 and 2, we used designs with 30 items, 30 trials and 5 items per trial (a parameter combination for which no BIBD exists). To find appropriate designs with these parameters, we used the design algorithm provided in Sawtooth Software’s *Lighthouse Studio* (Sawtooth Software, 2009), which randomly generates 1000 designs and then selects those with the best balance (see for more information). For Study 3, we used R to select a BIBD with 31 items, 31 trials and 6 items per trial.

**Presentation**

In the task, participants are shown the faces in a given trial, and must select the “best” and “worst” (e.g. “most attractive” and “least attractive”) faces from that trial. It is important that participants select exactly one best and one worst option, and do not choose the same face to be both “best” and “worst”.

In Studies 1 and 2 we presented our task online using Lighthouse Studio’s online survey service (Sawtooth Software, 2009) In Study 3, we used Qualtrics survey software (Qualtrics, 2018), using the heatmap question type set to allow a “best” and a “worst” response. We used custom validation to ensure that only one ‘best’ and one ‘worst’ response was selected. Of course, many other options exist; for example, Testable has a ranking format that can be extended to BWS trials, and any scripting language commonly used to build experiments (e.g. Java or Python) could certainly be used. The task could even be administered in pen-and-paper format.

**Scoring**

Once the data have been collected, the ‘best’ and ‘worse’ responses need to be converted to numeric scores. One simple scoring method is to subtract the number of times an item is selected as “best” from the number of times it is selected as “worst” across the entire task to give a score for that item. This “counts” score can then be normalized by dividing that total by the total number of trials in which the item appeared, giving a score that ranges from -1 to 1. Count scores are very easy to calculate, correlate highly with estimates derived from more sophisticated statistical models (Louviere, Flynn, & Marley, 2015, p. 21), and can be used to generate both group-level and participant-level scores. The support.BWS package for R contains functions to produce counts scores (see script S04 for a demonstration). The counts method is one of a number of algorithmic methods: see Hollis (2018) and Hollis and Westbury (2018) for an excellent primer on alternative methods and when they may be advantageous.

An elegant alternative approach is to use conditional logistic regression to model the probability that an item is selected as “best” or “worst” as a function of the items in a set (Louviere et al., 2015). An advantage of this regression approach is that it allows one to test statistical significance of differences in scores across items, unlike the count approach. However, limitation of this approach is that it cannot reliably estimate scores at the participant level if a participant always selects an item as best and never worst (or vice versa).

We used the counts method to derive BWS scores throughout Studies 1-3, as well as confirming that results do not change when using conditional logistic regression method to produce group-level scores in Study 1. We produced the group-level and individual scores using the scoring function provided in Lighthouse Studio (Studies 2 and 3) and using the support.BWS package in R (Study 3, see script S04), but note that the counts method could be implemented in Excel or even with a calculator and the conditional logistic regression model can be estimated in most statistical packages (e.g. SPSS, R or Stata).

**References**

Aizaki, H., Nakatani, T., & Sato, K. (2014). *Stated preference methods using R*: Chapman and Hall/CRC.

Di Paola, J. W., Seberry, J., & Wallis, W. (1973). A list of balanced incomplete block designs for r< 30. *Proceedings of the Fourth Southeastern Conference on Combinatorics, Graph Theory and Computing, Congressus Numerantium, 8*, 249-258.

Hollis, G. (2018). Scoring best-worst data in unbalanced many-item designs, with applications to crowdsourcing semantic judgments. *Behavior research methods, 50*(2), 711-729.

Hollis, G., & Westbury, C. (2018). When is best-worst best? A comparison of best-worst scaling, numeric estimation, and rating scales for collection of semantic norms. *Behavior research methods, 50*(1), 115-133.

Louviere, J. J., Flynn, T. N., & Marley, A. A. J. (2015). *Best-worst scaling: Theory, methods and applications*: Cambridge University Press.

Orme, B. (2005). Accuracy of HB estimation in MaxDiff experiments. *Sawtooth Software Research Paper,* http://www.sawtoothsoftware.com/download/techpap/maxdacc.pdf.

Qualtrics. (2018). Qualtrics. Provo, Utah, USA.

Sawtooth Software. (2009). Lighthouse Studio. Sequim, WA, United States: Sawtooth Software.
